# Beyond Algorithmic Accuracy: Field Readiness, Ecological Intelligence and Real-World Validation of Artificial Intelligence for Insect Pest Management

**DOI:** 10.3390/insects17101009

**Published:** 2026-09-30

**Authors:** Eda Budak Akbal, Erol Bayhan

**Affiliations:** 1 Vocational School of Agriculture, Dicle University, Diyarbakır 21280, Türkiye; 2 Department of Plant Protection, Faculty of Agriculture, Dicle University, Diyarbakır 21280, Türkiye; erolbayhan@gmail.com

**Keywords:** artificial intelligence, insect pests, integrated pest management, computer vision, smart traps, decision support

## Abstract

Artificial intelligence (AI) is rapidly reshaping insect pest identification, monitoring, and decision support, yet field maturity is often judged from algorithmic accuracy on curated or internally split datasets. Such evidence is insufficient for integrated pest management (IPM), where decisions depend on pest abundance, crop phenology, natural enemies, weather, economic thresholds, treatment costs, and asymmetric error consequences. This critical review synthesizes the literature on computer vision, automated counting, smart traps, Internet of Things (IoT), edge AI, multimodal sensing, population forecasting, uncertainty-aware AI, decision support, and precision intervention. We examine the laboratory-to-field generalization gap, small-object and long-tailed recognition, ecological incompleteness of pest-only datasets, sensor and trap bias, domain shift, calibration, human oversight, and operational constraints. We propose layered evaluation metrics spanning technical, quantitative, ecological, generalization, operational, decision, and outcome performance, and introduce a six-level Pest-AI Readiness Framework (PARF), from laboratory recognition to closed-loop precision management. We argue that future pest-AI systems should integrate visual observations with weather, crop phenology, trap history, and natural-enemy information, quantify uncertainty, support expert escalation, and undergo prospective external validation to generate timely, selective, and ecologically sound IPM decisions.

## Simple Summary

Artificial intelligence is increasingly used to identify and monitor insect pests, but high accuracy in laboratory images does not necessarily mean that a system will improve pest management in real fields. This review examines what is needed to move from insect recognition to reliable decision support. It considers field imaging, automated counting, smart traps, Internet of Things technologies, edge computing, population forecasting, uncertainty, beneficial insects, economic thresholds and precision intervention. We emphasize that useful systems must work across changing farms, seasons, devices and environmental conditions and must provide information that is meaningful for integrated pest management. We introduce a six-level Pest-AI Readiness Framework (PARF), ranging from laboratory recognition to closed-loop precision management. The framework distinguishes technical demonstrations from systems that have been validated for monitoring, forecasting, management decisions and intervention outcomes. Future progress will depend on combining artificial intelligence with ecological knowledge, independent field validation, transparent uncertainty, human expertise and economic evaluation. The goal should not simply be to recognize insects more accurately, but to support earlier, more selective and more sustainable pest-management decisions.

## 1. Introduction

Insect pests remain among the most persistent biological constraints on crop productivity and quality, and their management is fundamentally an information-and-decision problem. Growers and crop advisers must determine which organism is present, its abundance and life stage, whether the population is increasing, whether the crop is susceptible, whether natural enemies are exerting control and whether expected injury justifies intervention. The conceptual foundations of economic injury levels, economic thresholds and integrated pest management established this logic decades before modern AI [1,2,3,4]. Contemporary IPM frameworks have expanded the same principle by emphasizing prevention, monitoring, ecological regulation and the minimization of unnecessary pesticide exposure [5,6,7].

Conventional scouting, trap inspection and expert diagnosis remain indispensable, but they are labour-intensive, spatially sparse and often temporally coarse. These limitations have stimulated interest in machine learning (ML), deep learning (DL), computer vision, IoT sensing, unmanned aerial vehicles (UAVs), robotics and autonomous platforms. Broad agricultural reviews have documented applications ranging from crop classification and yield prediction to disease diagnosis, weed recognition and field robotics [8,9,10,11,12,13]. Plant-health studies likewise showed that high in-distribution performance can coexist with pronounced degradation outside the training domain, a lesson highly relevant to insect monitoring [14,15,16,17,18].

The insect-recognition literature has grown from handcrafted image features and classical classifiers to deep convolutional networks, attention mechanisms, one-stage detectors, instance segmentation and transformer-based models. Earlier machine-vision systems demonstrated that automated recognition could assist in taxonomic and crop-protection tasks [19,20,21]. Large datasets such as IP102 exposed the field to realistic long-tailed class distributions and fine-grained interspecific similarity [22], while subsequent studies developed residual, bio-inspired, multi-scale and fine-grained architectures [23,24,25,26]. In parallel, automatic trapping and field-monitoring studies moved recognition from static image collections toward continuous acquisition [27,28,29,30,31].

In this review, artificial intelligence (AI) is used as an umbrella term for computational systems that perform perception, prediction, reasoning, or decision-support functions relevant to pest management. Machine learning (ML) is treated as a subset of AI in which models learn statistical relationships from data; deep learning (DL) is a subset of ML based on multilayer neural networks, and computer vision denotes the image- and video-analysis task domain in which ML/DL methods are frequently implemented. These terms are therefore not used interchangeably.

Despite this progress, an explicit synthesis gap remains between common optimization targets in pest-AI research and what IPM requires for action. Recent reviews have provided valuable syntheses of insect classification and detection, computer-vision architectures, smart monitoring, and broader AI applications [26,32,33,34,35,36,37,38,39,40,41]. However, these studies are not consistently integrated across the complete evidence chain from biological sampling and quantitative monitoring to ecological interpretation, uncertainty, threshold-based decision support, intervention, and post-intervention outcomes. As a result, high performance on curated or internally split datasets can be discussed in the same language as operational readiness even though these claims require fundamentally different evidence. A further gap is the absence of a pest-management-specific readiness framework that jointly evaluates external validation, ecological context, resource constraints, uncertainty, human oversight, and demonstrated agronomic outcomes.

**Figure 1:**
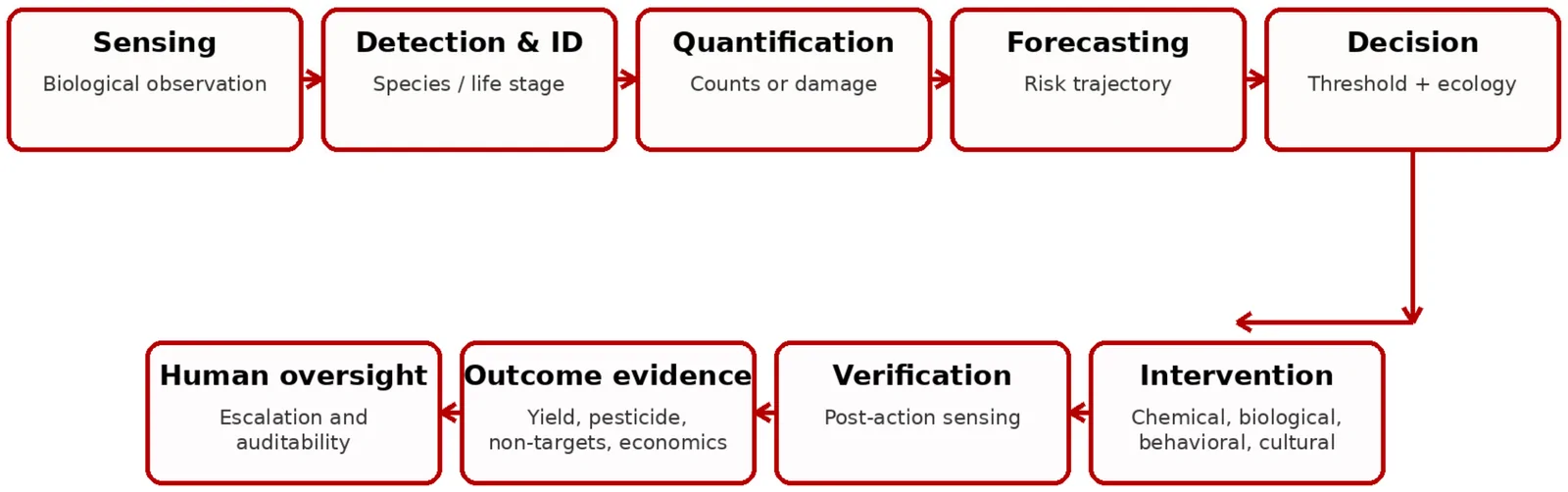
End-to-end pathway from biological sensing to accountable IPM action and outcome verification. Arrows indicate functional progression between stages rather than a mandatory linear sequence; human oversight and evidence requirements apply across stages.

To address these gaps, this review develops the concept of field-ready pest intelligence. Rather than organizing evidence around model families alone, we follow the complete management chain: sensing → detection → identification → quantification → forecasting → threshold interpretation → decision support → targeted intervention → post-intervention verification. We focus on the evidence required at each transition and on failure modes that may remain hidden by aggregate benchmark metrics. The review culminates in a six-level Pest-AI Readiness Framework (PARF), designed to distinguish laboratory demonstrations from systems supported by independent evidence for monitoring, prediction, management decisions, and intervention outcomes. This end-to-end management pathway is illustrated in Figure 1.

## 2. Scope and Analytical Framework of the Review

This article is a structured critical review rather than a formal systematic review or meta-analysis. The original evidence base was assembled iteratively from peer-reviewed primary studies, major datasets, foundational machine-learning literature, and high-relevance reviews, supported by topic-based searches and backward/forward citation chaining. Because this process was not prospectively registered as a systematic review, a database-specific search log and screening counts were not created at inception. During revision, we adopted PRISMA-S reporting principles [42] to make the search concepts, eligibility logic, supplementary search procedures, and limitations explicit. Supplementary Table S1 provides a platform-adapted, reproducible update-search protocol for Web of Science Core Collection, Scopus, IEEE Xplore, and ScienceDirect; it is presented as a reproducible update protocol rather than as a retrospective claim that the full corpus originated from one systematic query.

Eligibility was assessed against the end-to-end pest-management pathway rather than algorithm novelty alone. Studies were prioritized when they reported at least one of the following: biologically defined targets; field or trap acquisition conditions; independent validation across dates, sites, devices, seasons, or regions; quantitative pest-pressure outputs; beneficial-organism or crop-context information; forecasting or threshold interpretation; uncertainty or abstention; operational hardware evidence; or intervention/outcome evaluation. Purely methodological computer-vision papers were retained only when they supplied concepts necessary to interpret recurrent pest-AI architectures. Each included study was interpreted using seven questions: (1) what biological quantity is measured; (2) how representative are acquisition conditions; (3) how independent is the evaluation set; (4) does the output quantify pest pressure rather than identity alone; (5) are beneficial organisms and ecological variables represented; (6) is predictive uncertainty quantified; and (7) does the output support, trigger, or evaluate an intervention?

### 2.1. Search Reporting and Study Selection

The reproducible update-search protocol is organized around three concept blocks. The AI/engineering block includes ‘artificial intelligence’, ‘machine learning’, ‘deep learning’, ‘computer vision’, ‘convolutional neural network’, CNN, YOLO, ‘vision transformer’, transformer, ‘foundation model’, ‘multimodal learning’, and ‘edge AI’. The entomological block includes ‘insect pest’, ‘crop pest’, ‘agricultural pest’, ‘pest monitoring’, ‘pest detection’, ‘pest identification’, ‘pest classification’, ‘insect monitoring’, and ‘smart trap’. The management block includes ‘integrated pest management’, IPM, ‘economic threshold’, ‘decision support’, forecasting, ‘population prediction’, ‘precision pest management’, ‘biological control’, and ‘precision spraying’. Broad discovery combines the AI/engineering and entomological blocks, while focused searches add the management block. Supplementary Table S1 gives platform-adapted Boolean strings and documents citation chaining and targeted searches for benchmark datasets, smart-trap deployments, ecological decision-support studies, and foundational methods. For transparency, an auditable single ‘final search date’ cannot be assigned retrospectively to the original iterative evidence assembly because database-specific search logs were not prospectively retained. The Supplementary Table S1 protocol was finalized on 11 September 2026 as a reproducible procedure for future or update searches; it was not executed as one complete, auditable database-wide rerun during this revision. Thus, 11 September 2026 is a protocol-finalization date, not a completed-search date. Targeted revision-stage source verification and literature updating were instead performed for the evidence used in the PARF examples and for citation corrections. No lower date restriction is imposed on foundational IPM, statistics, or machine-learning sources when conceptually necessary. The protocol is restricted to English-language records and includes peer-reviewed original research, review articles, methodologically relevant conference proceedings, benchmark/dataset papers, and authoritative methodological guidance. Editorials, commercial webpages, patents, abstract-only records without sufficient methodological detail, duplicates, and records outside insect-pest management are excluded. If the protocol is run as a complete update in future work, database-specific search dates, hit counts, deduplication counts, and exclusions should be recorded prospectively rather than reconstructed after the fact.

Because the manuscript was originally conceived as a critical, problem-oriented review rather than a prospectively registered systematic review, database hit counts, deduplication counts, and full-text exclusion logs were not retained contemporaneously. These historical counts cannot be reconstructed reliably and have therefore not been invented retrospectively. Supplementary Figure S1 documents this limitation. The revised manuscript contains 153 references. Representative studies used for the PARF demonstration were selected purposively to span different biological targets, acquisition modes, validation designs, hardware constraints, and readiness levels. During the present revision, these exemplar studies were rechecked source-by-source for biological context, sampling duration, validation design, relevant quantitative results, and criterion-level PARF evidence; the resulting source locators and evidence-status codes are reported in Supplementary Table S2.

### 2.2. Descriptive Bibliometric Profile of the Cited Evidence Base

A quantitative descriptive bibliometric profile was prepared for the revised cited corpus (*n* = 153) using publication year and publication venue. Sixteen references (10.5%) were published up to 2015, 51 (33.3%) during 2016–2020, 42 (27.5%) during 2021–2023, and 44 (28.8%) during 2024–2026; thus, 86 of 153 references (56.2%) were published from 2021 onward. Among journal titles, *Computers and Electronics in Agriculture* was the most represented (23 references; 15.0%), followed by *Sensors* (8; 5.2%), *Agriculture* (7; 4.6%), *Frontiers in Plant Science* (6; 3.9%), and *Agronomy* (5; 3.3%). These descriptors characterize only the composition of the selected cited corpus; they are not estimates of field-wide prevalence, technology maturity, or readiness. Readiness conclusions in this review are therefore based on the study-level, criterion-specific evaluation in Section 14 and Supplementary Table S2 rather than on bibliometric composition. Supplementary Table S3 reports the descriptive bibliometric profile, and reference-level year/source data are supplied in machine-readable Supplementary Data S2.

### 2.3. Positioning Relative to Recent Reviews

Recent reviews have substantially advanced the literature on AI-based insect detection, classification, monitoring, and decision support, but they differ in scope and in the extent to which they address field validation, ecological context, operational constraints, uncertainty, and intervention outcomes [26,32,33,35,36,37,40,41]. Table 1 therefore positions the present review against closely related syntheses. Rather than duplicating architecture- or application-centred reviews, the present review integrates these dimensions across an end-to-end pest-management evidence chain and uses PARF to distinguish technical capability from progressively stronger evidence for monitoring, forecasting, decision support, intervention, and verified outcomes.

## 3. Sensing: The Biological Observation Enters the AI Pipeline

### 3.1. RGB Imaging and Acquisition Geometry

RGB cameras dominate pest-AI systems because they are inexpensive, compatible with smartphones and embedded platforms, and directly suited to modern computer-vision architectures. Yet “an insect image” is not a uniform data type. Handheld close-ups, sticky-trap images, pheromone-trap images, canopy photographs, microscope images, UAV imagery and robot-mounted video differ in scale, viewpoint, motion, background, illumination and biological sampling process. Early smartphone and mobile approaches already showed the promise of accessible field diagnosis [19], while later handheld deep-learning systems attempted multi-class wild pest monitoring under field conditions [43]. Trap-based systems simplify viewpoint and distance but introduce their own artefacts, including adhesive ageing, glare, dust, overlapping bodies and non-target captures [28,44,45].

Acquisition design is therefore part of the model design. Camera height changes apparent target size; illumination shifts texture and colour; lens quality affects edge detail; and trap colour alters both capture composition and image statistics. These interactions can create hidden shortcuts in which a model learns background or trap cues rather than insect morphology. Studies that experimentally manipulate acquisition conditions are especially valuable because they quantify deployment sensitivity rather than merely reporting another benchmark score. Recent work on wavelength-dependent insect detection demonstrates that detector performance can change substantially across lighting regimes [46], while sticky-trap research shows that trap colour itself can influence recognition performance [45].

### 3.2. Beyond RGB: Spectral, Thermal, Acoustic and Vibration Sensing

Not all pests are visually accessible. Hidden borers, subterranean insects, nocturnal activity and pre-symptomatic feeding motivate complementary sensing. Hyperspectral and multispectral imaging have long been investigated for crop stress and plant-health assessment, including stress signatures caused by pests and diseases [47,48,49]. Thermal imagery may reveal altered canopy temperature or transpiration, while acoustic and vibration sensing can capture the feeding or movement of cryptic insects. Remote sensing from UAVs extends spatial coverage and can sometimes detect infestation symptoms or host stress when direct insect imaging is impossible [50,51,52].

Beyond imaging, non-contact and contact sensing can capture insect presence through different physical signatures. Acoustic microphones and piezoelectric or vibration sensors can detect feeding, movement, or substrate-borne activity of concealed insects; optical beam-interruption and near-infrared (NIR) sensors can register passage, flight, or reflectance changes; capacitive and electric-field sensors can detect perturbations produced when insects enter or move near a sensing volume; and thermal sensors can identify temperature contrasts or plant physiological responses when direct visual resolution is insufficient. These modalities differ in spatial specificity, taxonomic resolution, susceptibility to environmental noise, and power demand. Consequently, modality choice should be linked to the biological question: direct taxonomic identification generally requires richer optical or spectral information, whereas event detection or activity monitoring may be achieved with lower-bandwidth physical sensors [53,54,55,56].

Recent examples illustrate the maturity gradient across these modalities. A dual-wavelength near-infrared system was validated during a four-week oilseed-rape field deployment and correlated with conventional yellow-water traps [54]. Electrical-field sensing has been field-tested for non-invasive insect activity and biomass estimation using CNN-based signal analysis [55], while capacitive sensing has recently been benchmarked as a low-power, non-optical approach for insect passage detection under controlled conditions [56]. These examples also show why sensor maturity should be judged separately from taxonomic resolution: a modality may be operationally robust for event detection or activity monitoring without yet supporting species-level diagnosis.

Multimodal sensing should not be adopted simply because additional channels improve cross-validation scores. Each sensor adds calibration requirements, data volume, power demand, synchronization complexity and cost. A field-ready design must show that the extra modality contributes information that changes a management decision. In this sense, sensor selection is an economic and ecological optimization problem as much as an engineering one.

## 4. From Recognition to Quantification

### 4.1. Classification and Fine-Grained Identification

Image classification remains useful for specimen or crop diagnosis, especially when the image is already centred on one organism. However, insect identification is intrinsically fine-grained: related taxa may differ by subtle morphological characters while the same species varies across sex, life stage, posture, damage condition and image scale. IP102 made these challenges explicit by providing more than 75,000 images across 102 pest categories with a naturally imbalanced distribution [22]. CNN-based and residual approaches improved representation learning for such tasks [21,24,57], and ensembles or attention-based strategies have further improved fine-grained discrimination [58,59].

The practical limitation of classification is that it assumes the organism has already been isolated. Field scouting rarely offers this convenience. A leaf may contain many aphids, mixed species, cast skins, predators and background structures. Thus, progress toward management requires localization and quantification, not only a species label.

The historical development of automated insect vision also demonstrates that the field did not begin with modern YOLO systems. Early work explored automatic identification of live moths and classical image-processing pipelines for pest recognition [60,61]. Later CNN studies showed that learned representations could improve insect detection and classification under increasingly complex image conditions [62,63], while benchmark initiatives supported fine-grained species discrimination [64]. These studies provide an important baseline: algorithmic sophistication has increased rapidly, but the core challenges of fine-grained morphology, background variation and representative sampling remain.

### 4.2. Object Detection and Small-Target Pest Recognition

Object detection simultaneously localizes and classifies targets. Two-stage detectors such as Faster R-CNN provide strong localization accuracy, whereas one-stage families such as YOLO emphasize speed and deployment efficiency [65,66]. Feature pyramids and multi-scale representations address variation in object size [67], and these design principles are particularly important for insect pests because targets may occupy only a tiny fraction of the image. Agricultural studies have used Faster R-CNN, YOLO variants and customized multi-scale networks for sticky traps, crop canopies and light-trap imagery [44,68,69,70,71,72].

Small-object detection remains one of the most persistent technical barriers. Aphids, thrips, whiteflies and early instars can become only a few pixels wide at practical working distances, and dense colonies produce severe overlap. Multi-scale Dense YOLO approaches, custom feature fusion and improved neck structures attempt to retain fine detail while maintaining real-time speed [73,74,75]. Transformer-based detectors may provide stronger global context but can require more computation unless compressed or distilled. The relevant comparison is therefore not simply mAP, but the joint distribution of small-object recall, latency, memory, power consumption and robustness.

### 4.3. Segmentation and Counting

For IPM, abundance is often the key variable. Counting can be derived from object detections, instance segmentation [76] or density maps. Detection-based counts are intuitive at low density but fail when bodies overlap; density-estimation approaches can perform better in crowded conditions but may lose individual identity. Li et al. [77] proposed a multi-branch CNN with density maps for aphid counting, while Gao et al. [78] combined detection and density estimation to switch between sparse and dense aphid distributions in sugar beet. More recent work has explored instance segmentation for wheat aphid infestation quantification [79], point-based counting for tiny insects [80] and microcontroller-oriented segmentation/counting models [81].

Counting error must be reported in units that map to management. Mean absolute error and root mean square error are more interpretable for abundance than classification accuracy, while infestation-class accuracy can be even more useful if scouting decisions are based on ordinal density categories. A model that consistently distinguishes “below threshold”, “near threshold” and “above threshold” may be operationally superior to one that estimates individual counts with lower average error but occasionally makes large threshold-crossing mistakes.

Continuous video and time-lapse monitoring introduce a temporal counting problem that is not captured by frame-level detection metrics. The same insect can appear in many consecutive frames, so summing per-frame detections can substantially overestimate abundance. Multi-object tracking (MOT), track association, and, where appearance changes or re-entry occurs, re-identification (Re-ID) are therefore necessary for temporal deduplication. Real-time insect tracking systems already demonstrate that track identity and residence time can be estimated from continuous camera streams [82,83,84]. For pest surveillance, evaluation should report identity switches, track fragmentation, duplicate-count rate, and count error after temporal deduplication rather than relying only on frame-level mAP.

Quantification in IPM should also include plant injury when counting individual insects is impractical or biologically indirect. Leaf miners, borers, mites, and other cryptic or dense pests may be better represented by damaged area, lesion or mine coverage, canopy stress, or ordinal infestation severity. Semantic/instance segmentation and anomaly-detection approaches can estimate the proportion of affected tissue, while spectral or thermal sensing can quantify stress signatures at larger spatial scales [47,48,49,50,51,52,79]. The relevant target should therefore be the management variable—individual abundance, damaged area, infestation class, or crop-stress severity—rather than insect bodies alone.

Recent detector studies reinforce the need to evaluate pest AI under realistic acquisition and sampling conditions. Systematic syntheses and trap-based experiments show that performance depends on target scale, trap background, insect density, camera configuration and deployment context [53,85,86]. Deep-learning systems have been developed for pheromone-trap counting, whitefly and thrips detection, multi-class field-pest detection, illustrating both the maturity of object detection and the persistence of small-object, crowding and background-confusion problems [68,87,88,89].

## 5. Datasets, Domain Shift and the Laboratory-to-Field Gap

### 5.1. Evaluation Splits, Dataset Shift and the Hidden Optimism of Random Splits

Most machine-learning pipelines assume that training and test samples are independent draws from the same distribution. Agricultural deployment violates this assumption because geography, cultivar, weather, device, farm management, season and pest phenology all change the data-generating process. The general dataset-shift literature distinguishes covariate shift, label shift and concept drift [90,91], while domain-adaptation research develops methods to transfer representations across related distributions [92,93]. These concepts are directly applicable to insect pest AI.

For pest monitoring, domain shift should also be described at the level of the sensing and sampling protocol. Trap colour, lure chemistry, camera optics, illumination, adhesive ageing, dust, trap height, and acquisition frequency can alter both image statistics and the biological community sampled. A model trained on blue sticky traps and deployed on yellow traps therefore encounters more than a cosmetic style shift: trap colour can change capture composition as well as background appearance [45]. Such a sensor/protocol shift cannot necessarily be solved by image style transfer because the underlying biological sampling distribution may also change.

A random split from one image campaign often allows near-duplicate backgrounds and acquisition conditions to occur in both train and test sets. This can substantially overestimate deployment performance. Stronger evaluation holds out acquisition dates, complete fields, seasons, devices or regions. The most convincing design is prospective: a frozen model is tested on data acquired later and independently. Such validation remains limited among the deployment-oriented pest-AI studies reviewed, even though broader ecological monitoring research increasingly emphasizes out-of-distribution testing and realistic field deployment [32,94].

Four validation designs should be distinguished explicitly. A random internal split samples training and test images from the same acquisition campaign and provides the weakest evidence for deployment. A temporally independent test holds out later acquisition dates or seasons from the same operational context. External field validation evaluates a frozen model in a distinct farm, device, trap type, region, or management setting. Prospective validation is stronger still because predictions are generated on future observations after the model and decision rules have been fixed, reducing opportunities for retrospective tuning.

Temporal generalization deserves separate treatment from spatial domain transfer. Within one species, seasonal temperature, developmental stage, sexual form, and phenotypic plasticity can alter size, colour, and wing morphology; aphid alate and apterous forms are a simple example. Over longer time scales, altered phenology, range shifts, population structure, and changing pest complexes can make historical training data progressively less representative. This is concept drift in an operational sense: the mapping between observed features, pest pressure, and management risk may change even when the sensor is unchanged. Field-ready studies should therefore report performance degradation across dates and seasons and define a monitoring/retraining policy when temporal drift is detected.

### 5.2. Long-Tailed Data, Rare Pests and Open-Set Recognition

Operational pest datasets are naturally imbalanced. Common taxa generate abundant training images, whereas invasive species, early outbreak stages and unusual life stages may be rare. Standard empirical risk minimization tends to favour frequent classes, precisely the opposite of what risk-sensitive surveillance may require. IP102 exemplifies long-tailed structure [22], and the long-tail learning literature shows why aggregate accuracy can mask poor minority-class performance [57,95]. Macro-averaged precision/recall, per-class sensitivity and rare-class recall should therefore accompany overall metrics.

Closed-set classifiers also assume that every encountered specimen belongs to a known training class. Real fields violate this assumption. Unknown non-target organisms, novel invasive pests or damaged specimens should not be forced into the nearest known category. Open-set recognition and abstention are thus highly relevant. Selective classification frameworks allow models to decline low-confidence predictions, which can then be routed to experts. This is especially important when a false identification could trigger pesticide treatment.

### 5.3. Data Augmentation, Synthetic Data and Self-Supervision

Data augmentation is widely used to increase variation in colour, scale, orientation and background. Mixup, CutMix and related techniques can regularize deep models, but augmentation should mimic plausible field variability rather than create biologically impossible images [96,97]. Synthetic imagery from generative models may help rare classes, yet it can also amplify annotation shortcuts and unrealistic morphology. Self-supervised learning offers an alternative by using large unlabeled image collections to learn transferable representations, reducing dependence on manually labelled pest data. Contrastive and masked-image approaches such as SimCLR and MAE established the general paradigm [98,99].

In insect science, large-scale foundation-style pretraining has begun to appear. Insect-Foundation introduced a million-image resource and transferable representations for insect understanding, and subsequent multimodal extensions explored vision-language learning [100,101]. These systems are promising for low-data taxa and taxonomic retrieval, but their agronomic value remains to be demonstrated under independent field deployment.

The smart-trap literature is also moving from isolated image classification toward connected sensing architectures. Embedded traps, IoT-enabled remote monitoring, edge inference and high-frequency camera systems have demonstrated practical routes for reducing inspection labour and increasing temporal resolution [30,102,103,104,105]. Field surveillance of economically important fruit flies and mobile decision-support tools further show how automated observations can be transmitted and interpreted in near real-time [31,106]. Laboratory benchmarking resources such as InsectCV can complement these field systems by providing reproducible trap-image evaluation [107].

## 6. Smart Traps, IoT and Edge AI

### 6.1. From Periodic Inspection to Continuous Data Streams

Automated traps can convert periodic manual sampling into high-frequency observations. A smart trap typically combines a biological capture mechanism, optical acquisition, on-device or cloud inference, communication, power and a data-management layer. Sun et al. [44] demonstrated an embedded in-trap detector using laboratory-simulated trap imagery generated from field-collected bark beetles, whereas field-deployed smart-trap systems have demonstrated repeated autonomous acquisition under natural conditions [27,30,31]. Camera-equipped trap reviews highlight substantial potential but also persistent constraints in cost, reliability, maintenance, standardization and validation [28]. The transition toward adaptive automated monitoring is also supported by online semi-supervised learning approaches that update pest-monitoring models as new observations become available [108].

Recent smart-trap systems increasingly integrate neural networks with low-cost embedded hardware and IoT communication. Zhao et al. [29] combined imaging, a microcomputer, a deep model and a server for automatic pest monitoring; Zhou et al. [109] demonstrated smartphone-based automated pest counting; Hinojosa-Dávalos et al. [110] reported a neural-network-guided selective smart trap; and Zarboubi et al. [111] integrated on-device YOLO detection, solar power and an IoT platform for codling moth monitoring. Such systems shift the engineering objective from maximal model size toward sustained, autonomous operation.

Automated monitoring has likewise evolved from static recognition toward counting, tracking and continuous surveillance. Vision-based systems have counted and recognized flying insects in intelligent-agriculture settings [112], trap-image studies have addressed automated moth detection [113], and machine-learning approaches have been used for aphid counting and classification [114]. More recent ecological camera systems extend this trajectory through real-time tracking, light-based nocturnal monitoring and open machine-learning pipelines [82,83,84]. Citizen-science imagery provides another route to scale insect identification, although label noise and domain heterogeneity require careful validation [115].

### 6.2. Communication Architecture and Event-Triggered Acquisition

Smart-trap reliability depends on how information moves between sensor, edge device, gateway, and cloud. Three common strategies are: (i) continuous transmission of raw images/video, which maximizes centralized flexibility but imposes high bandwidth and energy costs; (ii) edge inference with transmission of counts, thumbnails, or alerts, which reduces communication volume but places greater validation burden on the on-device model; and (iii) hybrid store-and-forward architectures in which local inference operates continuously while selected raw data are uploaded when connectivity and energy budgets permit. Communication protocols should be chosen according to coverage, payload size, latency tolerance, power budget, and maintenance constraints, and studies should report packet/image loss and offline behaviour in addition to nominal bandwidth.

Continuous monitoring does not require continuous image acquisition. Physical, optical, acoustic, or thermal triggers can activate the camera only when an insect crosses a beam, perturbs an electric/capacitive field, produces a characteristic signal, or enters a defined region of interest. Event-triggered capture can reduce redundant frames, storage, radio transmission, and power consumption, but trigger sensitivity becomes part of biological sampling: missed trigger events create an upstream false-negative mechanism that no downstream detector can recover. Trigger recall, false-trigger rate, wake-up latency, and energy saved should therefore be reported as system-level metrics [53,54,55,56,94].

### 6.3. Edge Deployment and Hardware-Tier-Aware Evaluation

Edge inference reduces bandwidth, latency and dependence on continuous connectivity, but imposes limits on memory, compute and energy. Lightweight architectures such as MobileNet and EfficientNet were developed specifically for resource-efficient vision [116,117,118], and quantization/pruning can further reduce deployment cost. In pest monitoring, ultra-lightweight camera-trap triggers and tiny segmentation/counting networks demonstrate the feasibility of edge processing [81,94]. Edge-oriented crop systems should report frames per second, latency, power draw, battery duration, memory footprint, thermal stability and communication volume alongside mAP.

The term ‘lightweight’ should always be tied to a hardware tier. TinyML-class microcontrollers (e.g., ESP32/STM32-class devices) generally require aggressively quantized, memory-constrained models and event-driven workloads; single-board computers such as Raspberry Pi-class devices can support larger CNNs at moderate frame rates; GPU-enabled edge modules such as Jetson-class devices can support substantially larger detectors and video pipelines; and smartphones occupy an intermediate but highly optimized deployment tier. A model that is real-time on a GPU edge module may be infeasible on a microcontroller because of RAM, flash, operator support, or thermal limits. Reporting should therefore pair model size and precision with target hardware, peak memory, latency/FPS, power draw, and sustained thermal behaviour.

A key principle is that sensing and inference cannot be evaluated separately. A 95% detector that causes battery failure after two days is less useful than a 91% detector that operates continuously for a month.

Similarly, cloud inference may be accurate but is unsuitable in fields with intermittent connectivity. Field readiness therefore requires system-level optimization rather than architecture-only optimization.

### 6.4. Sampling Validity Remains Biological

Digital automation does not remove the need for sampling theory. Trap catches are proxies whose relationship to crop infestation depends on lure chemistry, trap height, sex specificity, flight behaviour, weather and local population structure. Camera AI can count what the trap captures, but it cannot correct a biologically unrepresentative sampling process. This is a central reason why trap-level accuracy should not be equated automatically with crop-level risk. Field trials must establish the relationship between automated counts and independent crop scouting or damage measures.

## 7. Ecological Intelligence: From Pest Labels to IPM Context

### 7.1. Beneficial Arthropods and Asymmetric Error Costs

IPM is not a pest-only management. Predators, parasitoids and pollinators modify both pest trajectories and the environmental cost of intervention. Yet many public pest datasets are optimized for pest recognition rather than arthropod-community interpretation. From a management perspective, confusing a predator with a pest may be more consequential than confusing two non-target taxa, because the error can encourage unnecessary treatment and disrupt biological control. This motivates explicit reporting of a beneficial-insect false-positive rate and community coverage, not only conventional class-wise precision.

Recent work is beginning to incorporate pest-versus-beneficial discrimination and lightweight explainable models [119,120]. The next step is not merely to add beneficial classes but to connect their abundance to decision rules. Natural-enemy-to-pest ratios, parasitism rates and predator activity can alter economic thresholds in some cropping systems, and AI could help operationalize such context when validated ecological relationships are available.

Beneficial-organism information becomes actionable only when it is connected to the decision layer. In other words, Section 7.1 defines who else is present in the arthropod community, whereas Section 7.2 defines how that ecological context modifies whether a pest observation warrants intervention. This linkage is central to IPM because the same pest count may imply different actions when natural-enemy pressure, crop susceptibility, or expected population growth differs.

### 7.2. Economic Thresholds as the Missing Decision Layer

Economic injury levels and economic thresholds provide the conceptual bridge from monitoring to action [1,2]. However, many pest-AI studies stop at identification or counting. A decision-support system should transform observations into a recommendation using pest density, crop developmental stage, expected injury, crop value, treatment efficacy, treatment cost and, where scientifically defensible, natural-enemy activity. The distinction matters because the same pest count can imply different action at different crop stages or commodity prices.

Static thresholds are also vulnerable to climate and management variability. Temperature determines insect development rates, rainfall can modify survival, and crop phenology determines susceptibility. Classical degree-day and phenology models provide mechanistic structure that can be combined with data-driven forecasts. AI should therefore be viewed as a complement to, rather than a replacement for, ecological knowledge. Hybrid models can constrain predictions with biological relationships while learning residual patterns from the data.

### 7.3. From Ecological Observation to an AI-Supported Decision: An Illustrative Crop–Pest Pathway

A strawberry–two-spotted spider mite pathway illustrates the distinction between recognition and decision intelligence. Smartphone-based field counting has been demonstrated for spider mites, including deployment on common phones and spatial interpolation of pest observations [109]. In a management-oriented system, the image-derived count would not directly trigger treatment. It would first be interpreted with sampling location, crop stage, recent population trajectory, and, where available, predatory-mite observations and a locally validated action rule. The AI contribution is therefore conditional: it reduces observation burden and can improve spatial resolution, but the intervention recommendation remains bounded by crop-, region-, production-system-, and time-specific ecological evidence. This example also shows why a decision rule validated in one cultivar, climate, or production system should not be transferred automatically to another.

Direct evidence linking automated observations to validated management actions remains thinner than evidence for detection and counting. For example, smartphone-based spider-mite mapping in strawberry [109] demonstrates field quantification and spatial decision support, while greenhouse work integrating black-aphid detection with environmental sensing and population forecasting [121] moves closer to predictive intelligence. However, neither type of evidence should be treated as proof that a treatment threshold transfers unchanged across cultivars, regions, seasons, or production systems. Accordingly, PARF distinguishes monitoring or forecasting capability from locally validated threshold decisions and agronomic outcome evidence.

## 8. Crop-System Evidence and Representative Application Domains

### 8.1. Aphids and Other Colony-Forming Small Pests

Aphids illustrate why insect pest AI must progress from classification to quantitative and predictive intelligence. Their small body size challenges object detectors, colonies can range from isolated individuals to dense clusters, and their management often depends on both direct feeding and virus-transmission risk. Detection-only approaches may perform well at low density yet undercount heavily overlapping colonies. Density-map and hybrid counting approaches directly address this limitation [77,78], while wheat and sugar-beet studies increasingly combine detection, segmentation and infestation quantification [75,79]. These systems are scientifically valuable because the endpoint approaches the biological variable used in scouting. However, the next validation step is to test whether automated counts preserve rank order among fields, predict virus risk, and correctly classify plots relative to crop-specific intervention thresholds across seasons.

Aphid systems also highlight the importance of life-stage and natural-enemy information. Nymphs and adults differ in size and visibility; winged morphs can signal dispersal; mummies indicate parasitoid activity; and predators can occur in the same image. A detector trained only on aphids may therefore deliver a numerically accurate count while omitting the ecological context that determines whether control is needed. For this reason, aphid monitoring is an ideal test bed for PARF 4–5 systems that combine image-derived density with weather, crop phenology, virus-vector risk and beneficial-arthropod observations.

### 8.2. Sticky Traps, Pheromone Traps and Light Traps

Trap-based monitoring is one of the most mature routes to operational insect AI because image geometry is comparatively stable and the same device can acquire repeated observations. Pheromone-based work ranges from embedded in-trap detector prototypes developed with laboratory-simulated trap imagery [44] to long-duration remote field surveillance [31], and camera-equipped trap research has expanded across moths, fruit flies and mixed insect communities [28,30,70,113]. Light-trap systems broaden taxonomic coverage but create additional complexity from high diversity, wing posture, overlapping specimens and variable illumination [69,83]. Sticky traps constrain target distance but may accumulate debris and non-target captures, and recognition performance can depend on trap colour [45].

The operational value of automated traps depends on temporal reliability. A system that provides one high-quality image is not equivalent to one that produces daily counts for an entire season. Field papers should therefore report missing-image frequency, duplicate counting, image transmission failures, lure replacement schedules, adhesive degradation, lens contamination and battery state. These variables determine whether the resulting time series is suitable for forecasting. They also matter economically because a low-cost trap that requires weekly manual maintenance may provide little labour advantage over conventional scouting.

### 8.3. Mobile, Greenhouse and Open-Field Vision

Mobile diagnosis and open-field monitoring offer flexibility but face stronger domain shift than fixed traps. Smartphone and handheld systems have progressed from early vegetable pest recognition to CNN-based wild-environment monitoring and automated counting applications [19,43,109]. Greenhouse systems benefit from more constrained crop architecture but still encounter changing illumination, occlusion and dense small pests [27]. Open-field systems must cope with wind, variable canopy depth, sun–shade transitions and diverse backgrounds. These differences imply that a model trained for one acquisition regime should not be assumed transferable to another without explicit cross-device and cross-environment testing.

For mobile decision support, user behaviour becomes part of the sensing process. Farmers may photograph the most obvious insects, choose different distances, omit healthy reference plants or capture low-quality images. Training data collected by experts can therefore differ systematically from deployment data generated by non-specialists. Citizen-science imagery can increase taxonomic diversity [115], but label quality, geographic imbalance and photographer bias require robust curation. Practical mobile applications should include image-quality checks, guidance for acquisition and an abstention pathway when the target is too small or ambiguous.

## 9. Population Forecasting and Early Warning

Detection is retrospective: it reports what has already been observed. Management value increases when a system predicts whether pest pressure is likely to exceed an action threshold before damaging densities occur. Pest populations are driven by temperature, humidity, rainfall, host phenology, dispersal, natural enemies and management history. Classical statistical forecasting can model these relationships, while modern random forests, gradient boosting, recurrent neural networks and transformer architectures can capture nonlinear interactions and temporal dependence.

Forecasting approaches differ in what they assume and in the evidence they require. Degree-day and mechanistic phenology models are interpretable and data-efficient when development relationships are stable, but they may omit dispersal, natural enemies, or management effects. Classical autoregressive/time-series models can be strong baselines for repeated trap counts but assume a relatively stable temporal structure. Random forests and gradient boosting accommodate nonlinear weather–crop interactions but can lose explicit temporal ordering unless lagged features are engineered. Recurrent networks and temporal transformers can learn long dependencies, yet their flexibility increases data requirements and the risk of temporal leakage. Hybrid models that combine mechanistic development constraints with data-driven residual learning are attractive because they preserve biological structure while adapting to local patterns. Comparisons should therefore use temporally ordered splits, strong non-neural baselines, and prospective threshold-crossing outcomes rather than random cross-validation alone.

Evaluation should focus on management-relevant events rather than average prediction error alone. A model may achieve a low RMSE yet miss short outbreak peaks. Threshold-crossing sensitivity, false-alarm rate, lead time, calibration and the duration of useful warning are more appropriate. Palma et al. [122] evaluated a threshold-defined aphid-outbreak target in wheat at Coxilha, Rio Grande do Sul, Southern Brazil, using weekly observations collected from 2011 to 2019 (424 observations). The case-study split was chronological: initial fractions of the time series (40–80%) were used for training and the later observations for testing; with 80% training, the reported PBP accuracy was 95%. Because the monitoring interval was weekly and the method predicts the outbreak state at the subsequent monitoring sample, this constitutes a one-sampling-interval (approximately one-week) future-held-out prediction in that case study, rather than an intervention trial. Kapetas et al. [121] used a 44-day cucumber-greenhouse dataset and specified a seven-day black-aphid population forecast. Although leave-one-out evaluation was also discussed for machine-learning models, the ARIMAX analysis used a sequential split, with the initial 80% for training and the final 20% for testing, and reported an MSE of 75.61. Accordingly, the PARF 4 assignment for Kapetas et al. is tied specifically to this temporally ordered ARIMAX evaluation, not to the leave-one-out results. A prospective intervention trial is not a PARF 4 requirement; intervention and measured outcome evidence are evaluated at higher functional levels.

Climate change further increases the importance of external and temporal robustness. Range shifts, altered voltinism and novel pest complexes can produce conditions outside historical training data. Pest-AI models that assume stationarity may degrade as phenology and species interactions change. Climate robustness should therefore be considered a distinct axis of field readiness rather than a generic future limitation.

Operational efficiency is not only a hardware problem; model design must explicitly balance accuracy with inference cost. Lightweight architectures and carefully designed feature extractors can preserve useful pest-discrimination performance while reducing memory and computation requirements, which is important for continuous monitoring on field devices [123,124].

## 10. Explainability, Uncertainty and Human Oversight

### 10.1. Explainability: Why Saliency Alone Is Insufficient

Explainable AI (XAI) is frequently proposed as a mechanism for trust. Methods such as LIME, SHAP and Grad-CAM identify influential features or regions [125,126,127]. In pest recognition, explanation maps can reveal whether a model attends to diagnostic body structures or to spurious trap/background cues. However, visual plausibility does not establish causal or biological validity. Explanations should be evaluated against entomological knowledge and should address operational questions: what features drove the decision, which inputs drove a forecast, and how sensitive is the recommendation to plausible variation in pest counts or weather?

### 10.2. Calibration, Confidence and Uncertainty

Model confidence, probability calibration, and epistemic uncertainty should be distinguished. A softmax or detector confidence score is an internal ranking signal and is not automatically a calibrated probability. Calibration asks whether predictions assigned, for example, an 80% probability are correct approximately 80% of the time; it can be assessed using reliability diagrams, expected calibration error (ECE), or proper scoring rules such as the Brier score. Epistemic uncertainty concerns uncertainty in the model or parameters arising from limited or unrepresentative data and may increase under unfamiliar conditions. Temperature scaling, deep ensembles, Monte Carlo dropout, and conformal prediction provide complementary rather than interchangeable tools [128,129,130,131]. For pest surveillance, uncertainty is particularly important for rare species, novel backgrounds, temporal shift, and threshold-adjacent decisions.

### 10.3. Human Oversight, Abstention and Escalation

Human oversight should be specified as an operational rule rather than a general safeguard. A field-ready system should define when automated processing is permitted, when a prediction is withheld, and who receives an escalated case. Low-confidence observations, out-of-distribution inputs, rare or regulated pests, and decisions close to an economic threshold can be routed to an entomologist, extension specialist, or scout, while high-confidence routine observations are processed automatically. Escalation performance should be evaluated through coverage (the proportion of cases automated), selective risk/error among accepted predictions, expert workload, and the proportion of high-consequence errors intercepted. Human-in-the-loop design is therefore a risk-control mechanism, not evidence of incomplete autonomy.

## 11. Precision Intervention and Closed-Loop Management

The strongest sustainability claim for pest AI is that better information enables more selective intervention while maintaining crop protection. Precision intervention should not be equated with chemical spraying. Site-specific or variable-rate pesticide application, robotic or UAV-based delivery, and spot treatment are one pathway, but the same sensing-and-decision infrastructure can support the targeted release or redistribution of natural enemies, spatially selective pheromone or mating-disruption deployment, mass trapping, mechanical removal, localized sanitation, and cultural tactics such as trap-crop or hotspot management. The appropriate intervention depends on pest biology, crop system, available control options, and regulatory context. Precision agriculture and robotics research has established the feasibility of targeted sensing-and-actuation pipelines [132,133,134], but insect-pest applications remain less mature because insects are small, mobile, cryptic, and ecologically embedded.

A detection-triggered intervention should therefore be assessed across the full evidence chain: target detection, treatment placement, untreated pest escapes, non-target exposure, pesticide reduction, crop damage, yield protection and economic return. A detector that is accurate but causes excessive false-positive treatment may not improve sustainability. Conversely, a moderately accurate system may be valuable if its mistakes occur far below the threshold and its selective treatment substantially reduces pesticide volume.

Closed-loop management adds post-intervention verification. After treatment, the same sensing system should determine whether pest pressure declined and whether another intervention is necessary. This observation → risk estimation → intervention → verification cycle is routine in control engineering but uncommon in crop protection studies. PARF therefore reserves its highest readiness level for systems that demonstrate validated intervention and outcome verification rather than merely automated actuation.

Recent work has expanded deployment options across biodiversity monitoring, viticulture, cocoa, smart traps and low-compute platforms. Camera-trap systems can identify functionally diverse insects under complex scenes, while edge-compatible and lightweight detectors increasingly target on-device use [124,135,136,137,138,139]. Architectural research is also addressing long-tailed recognition, transformer-based fine-grained classification and scalable YOLO variants [140,141,142,143]. Broader reviews also extend this trajectory from recognition toward behavioural phenotyping and action-oriented pest management, emphasizing the need to connect computer-vision outputs with biologically and operationally meaningful endpoints [39,40].

Deployment diversity is also expanding. UAV-based detection has been explored for multi-class insect pests [144], while IoT-based intelligent pest-management systems combine sensing, communication and automated analysis to support precision agriculture [145]. Collectively, these studies reinforce the central argument of this review: field readiness emerges from the interaction between biological sampling, sensing hardware, model generalization, communications, human oversight and decision rules, not from the neural network alone.

## 12. Foundation Models, Multimodal AI and Language Interfaces

Vision transformers and foundation models are reshaping computer vision by learning transferable representations at scale [146,147]. DETR reframed object detection as a set-prediction problem [148], while large-scale self-supervision enables transfer to tasks with limited labels. For entomology, Insect-Foundation and multimodal insect-language resources suggest a path toward broader taxonomic coverage and interactive identification [100,101].

Current insect foundation models should therefore be interpreted cautiously. Large-scale pretraining can improve transfer and low-data recognition, but evidence of agricultural value requires testing on independent crop, trap, device, season, and geographic domains rather than only taxonomic retrieval or benchmark transfer. Performance should also be stratified for small pests, immature stages, damaged specimens, beneficial look-alikes, and open-set inputs. A foundation model that improves average identification while remaining poorly calibrated under field shift does not by itself advance decision readiness.

The most consequential future pest systems are likely to be multimodal rather than image-only. Visual detections can estimate identity and abundance; weather can indicate developmental rate; crop phenology can indicate susceptibility; historical trap counts can reveal trajectory; natural-enemy observations can modify expected growth; and management history can represent recent disturbance. Multimodal fusion can therefore shift AI from visual recognition toward ecological inference. The challenge is that every added modality introduces missing-data problems, calibration differences and new opportunities for spurious association.

Large language models (LLMs) can provide natural-language interfaces that translate technical model outputs into extension-style summaries, but they should not generate pesticide recommendations from unconstrained text priors. Recent prototypes have coupled YOLO detection with LLM-based interpretation [149], illustrating the interface potential. For safe agricultural deployment, recommendations should be grounded in locally validated thresholds, registered products, resistance-management constraints and region-specific regulations, with provenance retained for the underlying data and rules.

Reliability is the limiting issue for language interfaces. LLM outputs can be fluent while containing unsupported thresholds, obsolete pesticide information, or recommendations that are inconsistent with local labels and regulations. A safer architecture is retrieval- or rule-grounded generation in which the language model summarizes outputs from a validated detection/forecasting layer and cites the specific threshold rule, product registration, resistance-management constraint, and data timestamp used. Systems should preserve provenance, support refusal when required inputs are missing, and route high-consequence recommendations to human review. Language quality should never be treated as evidence of agronomic correctness.

Species-level discrimination remains a particularly stringent test of field readiness because closely related pests and beneficial or non-target taxa may co-occur on the same trap. Recent sticky-trap work on thrips and whiteflies demonstrates the practical difficulty of moving from coarse pest/non-pest recognition to species-resolved inference [150]. In parallel, automatic flying-insect monitoring and real-time tea-pest detection show the value of evaluating continuous counts, latency and population trajectories rather than image-level accuracy alone [151,152]. These studies support the view that model evaluation must be coupled to the biological quantity and management action that the sensing system is intended to inform. Microscopic tea-pest detection using an improved YOLOv8 network further illustrates the continuing emphasis on small-target detection under complex crop backgrounds [153].

## 13. Metrics for Field-Ready Pest AI

Conventional computer-vision metrics remain necessary but are insufficient. A layered framework better reflects the sequence from recognition to management outcome. Technical metrics evaluate discrimination and localization; quantitative metrics evaluate abundance; ecological metrics evaluate effects on beneficial organisms and life-stage coverage; generalization metrics test robustness across domains; operational metrics test deployability; decision metrics test threshold interpretation; and outcome metrics test whether the system produces agronomic or environmental benefit. The recommended metric layers are summarized in Table 2.

Metric definitions must be reported with enough detail to reproduce comparisons. For object detection, authors should specify the IoU threshold used for mAP and whether results refer to mAP@0.5, mAP@0.75, or COCO-style mAP@[0.5:0.95]. MAPE should be avoided or explicitly stabilized when true counts are zero or close to zero. Metric choice should also reflect asymmetric error costs: near an economic threshold a false negative may be more costly than a false positive, whereas for a beneficial organism the reverse may hold. Cost-sensitive metrics, decision curves/net benefit, rare-class recall, calibration error, out-of-distribution performance, abstention performance, and temporal degradation therefore complement conventional accuracy. A calibrated probability can be combined with treatment cost and expected damage; an uncalibrated confidence score cannot be interpreted economically.

## 14. Pest-AI Readiness Framework (PARF)

To distinguish technical demonstrations from operational pest-management systems, PARF uses six functional readiness levels but is not intended as a rigid technological staircase. A system may follow different development pathways according to its intended function: for example, an expert-scouting decision-support tool may reach decision-level readiness without automated continuous monitoring, whereas a local closed-loop controller may operate without long-horizon population forecasting. PARF therefore combines the six functional levels with five evidence dimensions—biological/sampling validity, model generalization, operational reliability, decision integration, and outcome evidence. A dimension may be designated ‘not applicable’ when it is genuinely outside the intended use, but evidence that is essential to the claimed function cannot be compensated for by strength in another dimension. The six levels and their minimum evidence requirements are summarized in Table 3.

For formal future applications of PARF, assignments should be made by at least two independent evaluators using the same evidence-extraction sheet, with disagreements resolved by consensus and the rationale reported. This two-evaluator procedure is a recommended implementation rule; it was not prospectively applied to the original evidence extraction for this critical review. During the present revision, the illustrative assignments were re-audited source-by-source against the explicit minimum criteria and exact source locations reported in Supplementary Table S2. That table distinguishes M (criterion explicitly established), NR (the source explicitly does not report the item), NE (not established from the source-level evidence located in this review; this does not imply that the original study necessarily lacked the evidence), and N/A (genuinely not applicable to the intended system function). The assigned level is the highest functional claim for which every required applicable criterion is established; desirable evidence strengthens confidence but cannot compensate for an unmet or unverified minimum criterion. PARF does not prescribe one universal minimum number of days for field validation because biologically meaningful duration differs between a short trap-validation task and a seasonal forecasting claim. Instead, the study must justify that the evaluation spans the monitoring or decision window implied by the claim and should report exact duration, independent dates/sites/seasons and frozen-model status. Calibration evidence should include a reliability assessment or proper scoring metric when probabilistic decisions are made, and abstention/escalation rules should be evaluated where high-consequence uncertainty exists. Every assignment is context-bounded to the pest–crop–region–device/acquisition configuration and cannot be transferred automatically to a new context. Representative source-audited applications are summarized in Table 4.

## 15. Research Priorities for the Next Generation of Pest AI

Research priorities can be grouped into four evidence gaps. First, validation should be external and prospective by design, using farm-, season-, device-, trap-, and region-level holdouts with transparent provenance and open benchmark protocols. Second, datasets should be ecologically complete enough for the intended decision, including life stage, crop stage, damage symptoms, beneficial organisms, and uncertainty where relevant. Third, engineering should be resource-aware, reporting hardware, latency, memory, power, communication, trigger performance, uptime, and maintenance rather than model accuracy alone. Fourth, evaluation should move toward action-oriented endpoints: abundance and damage error, threshold crossing, outbreak lead time, calibrated uncertainty, labour and pesticide savings, non-target effects, yield protection, economic return, and post-intervention verification.

Climate robustness and multimodal ecological fusion cut across these priorities. Temporal transfer should be tested as phenology, pest ranges, and species interactions change, while imagery should be combined with weather, crop phenology, trap history, natural enemies, and management history only when the additional variables improve a prespecified decision. Closed-loop trials remain the strongest test because they connect observation, risk estimation, selective intervention, and verified outcomes.

## 16. Data Quality, Reproducibility and Governance

Data quantity alone is a poor proxy for dataset value. For insect pest management, high-value datasets document where, when and how each image was collected; the crop and cultivar; trap or camera type; phenological stage; pest life stage; annotation protocol; and whether multiple images derive from the same sampling unit. Without provenance, researchers cannot construct truly independent validation splits or diagnose model failure. Public benchmarks such as IP102 accelerated algorithm development [22], but future benchmarks should go further by publishing farm-, season- and device-level metadata that permit predefined external validation.

Annotation quality is particularly important for small insects. Bounding boxes become ambiguous when targets overlap or occupy only a few pixels, and disagreements among annotators may exceed the performance differences between competing models. Expert taxonomic verification is essential for morphologically similar taxa, while uncertain specimens should be labelled as uncertain rather than forced into a class. Inter-annotator agreement, adjudication procedures and life-stage definitions should be documented. For counting tasks, the reference standard should specify whether partially visible insects, exuviae, dead specimens and overlapping individuals are included.

Reproducibility requires more than publishing source code. Studies should specify training/validation/test splits, random seeds, image resizing, augmentation, confidence and non-maximum-suppression thresholds, hardware and model checkpoints. Dataset leakage should be actively prevented by grouping images from the same trap, plant, video sequence or acquisition date before splitting. These practices are especially important in agricultural imagery because consecutive frames and repeated trap images can be visually near-identical. Reproducible external splits would make progress more meaningful than repeated re-tuning on the same benchmark.

For reproducibility, manuscripts should label validation designs consistently: ‘random internal’ for same-campaign shuffled splits; ‘temporally independent’ for later dates/seasons in the same operational setting; ‘external field’ for a distinct farm/device/trap/region; and ‘prospective’ when a frozen model is evaluated on observations collected after model finalization. These labels should not be used interchangeably because they support different strengths of field-readiness claim.

Governance also becomes important as systems move toward automated recommendations. Pest images may be linked to farm locations, production records and management history. Data-sharing agreements should therefore define ownership, access and retention, while decision-support systems should preserve provenance for recommendations. If an AI system recommends treatment, users should be able to trace the recommendation to the detected pest pressure, environmental inputs, threshold rule and model version. This auditability is a practical requirement for accountable agricultural AI.

## 17. Adoption, Economics and Implementation Barriers

The adoption question is not whether AI is technically impressive, but whether it provides a net benefit relative to existing scouting. Automated monitoring can reduce repetitive labour and increase temporal resolution, but cost includes cameras, traps, communication, data services, batteries, replacement parts, calibration and maintenance. Preti et al. [28] emphasized that the economics of camera-equipped traps are central to large-scale adoption. Resource-efficient models and low-cost IoT architectures may reduce hardware costs [111,116,117,145], yet a complete economic assessment must include human time spent correcting errors and maintaining the system.

The value of information is crop- and pest-specific. In a high-value crop with a rapidly developing pest, one day of earlier warning may justify substantial monitoring cost. In a low-value crop with a wide treatment window, the same system may not be economical. Similarly, automatic identification has limited value if intervention thresholds are absent or if growers already apply prophylactic treatments independent of scouting. Economic evaluation should therefore compare the AI-supported management pathway with a realistic baseline, reporting labour savings, avoided sprays, pesticide volume, yield protection and return on investment rather than hardware price alone.

Adoption also depends on perceived reliability and fit with work routines. Farmers and advisers need to understand when the system can be trusted and when expert review is required. Calibration and human-in-the-loop design can support this expectation management [128,130]. Interfaces should communicate uncertainty in actionable terms rather than raw neural-network scores. For example, “high confidence that the field is below threshold” is more useful than a species probability if the decision objective is whether to spray.

A practical adoption decision can be represented as a gated pathway: (1) define the crop–pest decision and the cost of current scouting; (2) confirm that the AI system measures a variable linked to a locally valid management rule; (3) verify external field performance and uncertainty on the intended hardware; (4) quantify total ownership cost, including sensors, connectivity, maintenance, expert review, and model updating; (5) compare the AI-supported pathway with realistic standard practice using labour, pesticide use, yield, non-target effects, and net return; and (6) adopt at scale only when the expected net benefit remains positive under plausible failure and maintenance scenarios. This decision tree prevents inexpensive hardware or high accuracy from being treated as sufficient evidence of economic adoption.

Finally, implementation should avoid creating a false dichotomy between autonomous AI and human scouting. The most realistic near-term architecture is complementary: automated systems provide continuous high-frequency surveillance, while humans verify exceptions, diagnose novel organisms and make high-consequence decisions. This division of labour can increase sampling intensity without requiring complete automation. PARF therefore treats human oversight as a component of mature decision intelligence rather than evidence that a system is incomplete.

## 18. Discussion

The literature now contains sophisticated classifiers, detectors and segmenters, but technical capability is advancing faster than evidence of management value. Insect computer vision has clearly moved beyond proof of concept: large benchmarks exist, small-object detectors have improved, smart traps can operate autonomously, edge models can run on resource-constrained devices, and multimodal/foundation approaches are emerging [22,28,29,94,100]. The limiting question is increasingly not whether insects can be recognized, but whether performance survives the heterogeneity of agricultural production systems.

This distinction has practical consequences for research design. For computer scientists, a small reduction in benchmark mAP may be worthwhile if it yields better calibration, lower power draw or stronger external robustness. For entomologists, dataset construction should prioritize biologically meaningful labels and independent sampling designs rather than simply maximizing image counts. For agronomists and economists, evaluation must connect automated observations to threshold decisions, pesticide savings, labour savings and yield protection. These disciplines are interdependent; none can establish field readiness alone.

The PARF is intentionally demanding. Many current studies will fall within levels 1–3, and this should not be interpreted as a criticism. Recognition, field detection and repeated automated monitoring are necessary foundations. The purpose of readiness staging is to prevent early technical success from being described as complete decision intelligence. Higher levels require prospective evidence that the system forecasts relevant risk, quantifies uncertainty, preserves ecological context and improves management outcomes.

A likely future architecture is distributed: a compact edge detector operating continuously at the trap or crop; a cloud or gateway model integrating weather and historical counts; a foundation model assisting rare-species identification; and a decision layer applying local IPM thresholds and management constraints. Such systems may be more powerful than one monolithic model, but validation also becomes more complex. Uncertainty introduced at detection propagates into forecasts and recommendations, and it should be quantified rather than hidden.

The ultimate benchmark for pest AI is therefore not a leaderboard. It is whether a grower, scout or decision-support system can act earlier, more selectively and with less ecological and economic risk. Research that demonstrates this connection will be more consequential for sustainable crop protection than repeated marginal improvements in internally split image datasets.

## 19. Conclusions

High algorithmic accuracy is not synonymous with readiness for insect-pest management. Field-ready systems must preserve performance across relevant farms, seasons, devices, trap or sensing protocols, and biological conditions, while measuring variables that map to IPM decisions. The most important transition is therefore from recognition-oriented AI to decision-oriented pest intelligence that combines abundance or damage, crop and weather context, natural enemies where relevant, and locally defensible thresholds.

PARF formalizes this transition as six functional readiness levels assessed across biological validity, model generalization, operational reliability, decision integration, and outcome evidence. The framework is evidence-based and context-bounded rather than architecture-based or obligatorily linear: a simpler model with prospective external validation and demonstrated management value can be more field-ready than a state-of-the-art model evaluated only on a curated benchmark. Mature systems should also quantify uncertainty, allow abstention, preserve human escalation for unfamiliar or high-consequence cases, and report the hardware and communication conditions under which performance was achieved.

The strongest future evidence will come from prospective studies that connect sensing to quantitative pest pressure, forecast or threshold interpretation, selective intervention, and post-intervention verification, while measuring pesticide use, biological-control preservation, labour, yield, non-target effects, and economic return. The central question for pest AI is thus not only whether an insect can be recognized, but whether the resulting information improves the subsequent management decision in a reliable, economically defensible, and ecologically sustainable way.

## Figures and Tables

**Table 1:** Positioning of the present review relative to closely related recent syntheses.

Reference	Primary Emphasis	Field/Decision Emphasis	Framework Contribution Relative to PARF
Li et al. [26]	Systematic review of deep-learning insect classification/detection from field images	Field imaging and smart pest management; mainly perception-focused	Provides architecture/task synthesis; does not stage readiness from sensing through verified intervention
Høye et al. [32]	Computer vision and deep learning across entomology	Broad ecological monitoring perspective	Establishes transformative potential but not a pest-IPM readiness rubric
Guo et al. [33]	Pest detection and recognition algorithms	Algorithmic overview	Benchmark/architecture oriented; limited decision and outcome staging
Amiri and Bandani [35]	AI for pest identification and decision support	Strong sustainable crop-protection orientation	Closest conceptual neighbour; PARF adds explicit evidence levels, operational constraints, uncertainty, and outcome verification
Kariyanna et al. [36]	AI applications in insect-pest management	Broad application review	Application taxonomy rather than operational readiness classification
Leybourne et al. [37]	Integration of AI into pest-monitoring schemes	Entomological, management, and computational perspectives	Highlights implementation barriers; PARF formalizes evidence requirements across readiness claims
Li et al. [40]	From detection to action in integrated pest/invasive plant management	Action-oriented AI perspective	PARF adds staged evidence, calibration/human oversight, and context-bounded readiness
Chakrabarty et al. [41]	AI in insect-pest identification	Identification-focused review	PARF extends beyond identification to forecasting, decisions, intervention, and measured outcomes
Present review	End-to-end field readiness for insect-pest AI	Biological sampling → quantification → ecology → uncertainty → decision → intervention → verification	Six functional readiness levels plus multidimensional evidence domains and context-bounded assignments

**Table 2:** Layered metrics for evaluating field-ready pest AI.

Layer	Recommended Metrics	Why It Matters	Failure Hidden by Accuracy
Technical	Precision, recall, F1; mAP@IoU threshold(s) and mAP@[0.5:0.95] where relevant; IoU; rare-class recall	Recognition/localization quality	Strong mean score but weak rare-class or small-object recall
Quantitative	MAE/RMSE; count bias; infestation-class accuracy; MAPE only when reference counts are safely above zero	Links AI to pest pressure	MAPE becomes unstable or undefined at zero/near-zero counts
Ecological	Beneficial false-positive rate; life-stage sensitivity; community coverage; damage-severity agreement	Preserves IPM context	Predators/parasitoids treated as pests or injury poorly represented
Generalization	Cross-date/farm/season/device/trap/region performance; temporal degradation; out-of-distribution detection	Tests spatial and temporal robustness	Random splits conceal domain/sensor/concept shift
Operational	Latency; FPS; peak memory; model size; power; bandwidth; uptime; trigger recall; maintenance interval	Determines deployability on stated hardware	Accurate model cannot operate reliably or misses events upstream
Uncertainty	Reliability curve; ECE; Brier score; coverage vs. selective risk; abstention/OOD performance	Determines whether confidence can support risk-aware escalation	Overconfident errors or excessive expert workload
Decision	Threshold-crossing sensitivity/specificity; false-alarm rate; lead time; decision calibration; decision-curve/net benefit	Tests management relevance	Good regression but poor treatment decisions
Outcome	Pesticide reduction; biological-control preservation; yield protection; labour saving; economic return; non-target impact	Tests real-world value	Automation without agronomic or ecological benefit

**Table 3:** Operationalized six-level Pest-AI Readiness Framework (PARF).

Level	Functional Designation	Required Evidence (Minimum)	Desirable Evidence and Context/Transition Rule
PARF 1	Laboratory recognition	Curated or controlled images; documented labels; internal validation with leakage control	Calibration and rare-class reporting. Claim limited to represented taxa, image protocol and dataset.
PARF 2	Field detection	Uncontrolled field/trap images plus an independent acquisition date at minimum; test data must be separated from training by a biologically meaningful grouping variable (date/site/device/trap/region).	External site/device/season test; OOD analysis; prespecified frozen-model evaluation. Field readiness is specific to the pest-crop-region-device/acquisition context.
PARF 3	Automated monitoring	Repeated acquisition; validated counting/quantification; a duplicate-control strategy where repeated observations can re-observe the same individual; uptime/maintenance evidence; deployment spanning at least one complete decision-relevant monitoring window, or an explicit biological justification for a shorter interval.	Season-scale operation; communication/trigger reliability; comparison with manual scouting. Duration must match the intended claim and pest biology; report total deployment days, sampling interval, missing-data frequency and whether the period captures the relevant population/decision cycle.
PARF 4	Predictive intelligence	Temporally ordered prediction with environmental and/or trajectory inputs; prespecified prediction target and forecast horizon; prospective or strictly future-held-out validation.	External-season/region forecasting; calibrated risk; threshold-crossing lead time. A prospective intervention trial is not required at this level.
PARF 5	Decision intelligence	Validated decision rule or threshold; ecological context where relevant; calibrated uncertainty/abstention with reliability evidence (e.g., reliability curve, ECE/Brier score or equivalent); explicit human-escalation rule for high-risk or ambiguous cases.	Prospective comparison with standard scouting/decision practice; net-benefit analysis. Readiness applies only where the underlying threshold/rule is locally defensible.
PARF 6	Closed-loop precision management	Selective intervention triggered by the system plus post-intervention verification and measured agronomic/ecological outcome.	Replicated commercial-scale trial; economic return; non-target outcome. The claim is bounded to the tested crop, pest, production system, region, hardware and control tactic.

**Table 4:** Source-audited illustrative application of PARF to representative published systems. Assignments reflect evidence explicitly established for the cited study; unverified minimum criteria cap the assigned level rather than being treated as satisfied.

Study	Biological/Operational Setting	Source-Audited Validation/Deployment Evidence	Indicative PARF	Evidence-Based Ceiling
Wu et al. [22] (IP102)	Multi-class pest-image benchmark	75,222 images/102 classes; documented train/validation/test benchmark split (dataset description/split; Table 1).	1	Recognition benchmark only; no operational field acquisition or monitoring outcome.
Sun et al. [44]	Embedded in-trap Dendroctonus valens detector	Field-collected bark beetles were randomly placed into the trap collection cup in the laboratory to simulate field traps; internal detector evaluation and embedded runtime were reported (In-trap image acquisition; Experiments and results).	1	PARF 2 requires uncontrolled field/trap acquisition plus an independent date/site/device/trap/region grouping. That minimum evidence is not established; the prior PARF 2 assignment was therefore downgraded.
Geissmann et al. [30] (Sticky Pi)	High-frequency autonomous insect camera trap	Images every 20 min; 10 Sticky Pis deployed in two berry fields for 11 weeks; cross-frame insect matching/tracking; approximately 2 weeks device autonomy without solar (Results and field-deployment methods in the original study).	3	Repeated validated monitoring, temporal duplicate control and operational duration are demonstrated; no pest-risk forecast or validated decision threshold.
Diller et al. [31]	Remote surveillance of economically important fruit flies	Multi-country field deployment; 60–257 d continuous operation; >300 Israel/South Africa images not used in training were checked against entomologist classification; field-validation precision ranged from 86% to 97%; communication/upload performance was reported (field-evaluation methods and Results in the original study).	2	Strong field and operational evidence, but source-level extraction did not establish a temporal duplicate-control rule required by PARF 3; conservatively capped at PARF 2.
Zhou et al. [109]	On-demand smartphone monitoring of two-spotted spider mite in strawberry	Field testing on Samsung Galaxy S10e and LG Stylo 6; comparison with manual counting; motile/egg counting accuracy 78.1%/75.3%; GPS-linked spatial interpolation (Abstract; field-evaluation results).	2	Field quantification is demonstrated. A repeated automated monitoring window, uptime/maintenance evidence and PARF 3 temporal acquisition requirements are not established for this on-demand smartphone design.
Palma et al. [122]	Threshold-defined aphid outbreak prediction in wheat	Coxilha, Rio Grande do Sul, Southern Brazil; weekly sampling from 2011 to 2019, 424 observations; prediction of the subsequent monitoring sample; chronological initial-fraction training/later testing; PBP accuracy 95% with the 80% training split (case-study methods and results in the original study).	4	Strictly future-held-out time-series evaluation supports predictive intelligence; a treatment trial is not required for PARF 4.
Bjerge et al. [82]	Real-time in situ insect camera system	Summer 2020 deployment for 98 d; 2994 insect tracks; 504 manually verified tracks across 10 d; average precision 89%; individual tracking and daily server upload (Abstract/Methods/Results).	3	Repeated acquisition, validated track-level quantification and individual tracking support PARF 3; no pest-management forecast or threshold decision.
Guo et al. [151]	Flying vegetable pests on yellow sticky traps	RGB camera and yellow sticky traps installed in vegetable fields; automatic image acquisition; mAP 84.22%, aMAE 0.422 and aMSE 1.126 (Abstract; experimental results).	2	Field detection/counting is supported, but monitoring duration, duplicate-control logic and uptime/maintenance evidence required for PARF 3 are not established in the evidence audited here.
Kapetas et al. [121]	Black-aphid detection and forecasting in cucumber greenhouse	Five sticky traps in a cucumber greenhouse; 220 images over 44 d; seven-day forecast target; ARIMAX evaluated with sequential initial 80% training/final 20% testing; MSE 75.61 (greenhouse-data and population-prediction methods and results in the original study).	4	PARF 4 is supported specifically by the temporally ordered ARIMAX evaluation. Leave-one-out evaluation of other ML models is not used as evidence for future-held-out readiness.

## Data Availability

No primary experimental or field data were generated. This critical review generated secondary structured evidence-extraction data and descriptive bibliometric data. The criterion-level PARF evidence matrix is provided in Supplementary Table S2 and as machine-readable Supplementary Data S1 (CSV). Reference-level publication-year/source data supporting the descriptive bibliometric profile in Table S3 are provided as Supplementary Data S2 (CSV).

## Supplementary Materials

The following supporting information can be downloaded at: https://www.mdpi.com/article/10.3390/insects17101009/s1, Table S1: platform-adapted reproducible update-search protocol; Figure S1: evidence-identification and transparency workflow; Table S2: criterion-level source-audited evidence matrix for illustrative PARF assignments; Table S3: descriptive bibliometric profile of the revised cited corpus; Supplementary Data S1: machine-readable PARF evidence matrix (CSV); and Supplementary Data S2: reference-level year/source data supporting Table S3 (CSV).
