# Supplementary material for: Beyond Algorithmic Accuracy: Field Readiness, Ecological Intelligence and Real-World Validation of Artificial Intelligence for Insect Pest Management
Source: Insects. 2026 Sep 30;17(10):1009. doi: 10.3390/insects17101009 (PMC13704983; doi:10.3390/insects17101009)
Supplement: Supplementary file 1 [file insects-17-01009-s001.zip › insects-4560668-supplementary.pdf]

Supplementary Information

Beyond Algorithmic Accuracy: Field Readiness, Ecological Intelligence and Real-World Validation of Artificial Intelligence for Insect Pest Management

Eda Budak Akbal and Erol Bayhan

Transparency Note: This manuscript is a structured critical review, not a prospectively registered systematic review. The original evidence base was assembled iteratively; therefore, historical database hit counts, deduplication counts, and full-text exclusion logs were not retained and are not reconstructed retrospectively. Table S1 is a reproducible update-search protocol developed during revision; it was not executed as one complete database-wide rerun, and its finalization date is not presented as a completed search date. Targeted source verification and source-specific literature updating were performed for the revised PARF evidence audit. The materials below provide transparent search and evidence-extraction documentation without implying that the full cited corpus arose from one exhaustive systematic query.

Table S1. Platform-adapted reproducible update-search protocol

The three concept blocks are combined as (AI/engineering) AND (entomology), with the management block added for focused decision-oriented searches. Because the original critical-review evidence base was assembled iteratively and database-specific logs were not retained prospectively, no unverifiable retrospective hit, deduplication, or exclusion counts are supplied. The protocol was finalized on 11 September 2026 for reproducible future/update use but was not executed as a single complete database-wide rerun during this revision. If run in future work, searches should be limited and documented according to the stated language/document-type criteria, and database-specific search dates and counts should be recorded prospectively.

Platform / route	Broad discovery string	Focused management string	Reporting note
Web of Science Core Collection	TS=("artificial intelligence" OR "machine learning" OR "deep learning" OR "computer vision" OR CNN OR YOLO OR "vision transformer" OR transformer OR "foundation model" OR "multimodal learning" OR "edge AI") AND TS=("insect pest" OR "crop pest" OR "agricultural pest" OR "pest monitoring" OR "pest detection" OR "pest identification" OR "pest classification" OR "insect monitoring" OR "smart trap")	TS=("artificial intelligence" OR "machine learning" OR "deep learning" OR "computer vision" OR CNN OR YOLO OR "vision transformer" OR transformer OR "foundation model" OR "multimodal learning" OR "edge AI") AND TS=("insect pest" OR "crop pest" OR "agricultural pest" OR "pest monitoring" OR "pest detection" OR "pest identification" OR "pest classification" OR "insect monitoring" OR "smart trap") AND TS=("integrated pest management" OR IPM OR "economic threshold" OR "decision support" OR forecasting OR "population prediction" OR "precision pest management" OR "biological control" OR "precision spraying")	Use for multidisciplinary discovery; document index coverage and search date when rerun.
Scopus	TITLE-ABS-KEY(("artificial intelligence" OR "machine learning" OR "deep learning" OR "computer vision" OR CNN OR YOLO OR "vision transformer" OR transformer OR "foundation model" OR "multimodal learning" OR "edge AI")) AND TITLE-ABS-KEY(("insect pest" OR "crop pest" OR "agricultural pest"	TITLE-ABS-KEY(("artificial intelligence" OR "machine learning" OR "deep learning" OR "computer vision" OR CNN OR YOLO OR "vision transformer" OR transformer OR "foundation model" OR "multimodal learning" OR "edge AI")) AND TITLE-ABS-KEY(("insect pest" OR "crop pest" OR "agricultural pest"	Record document-type and language filters when rerun.

Platform / route	Broad discovery string	Focused management string	Reporting note
	OR "pest monitoring" OR "pest detection" OR "pest identification" OR "pest classification" OR "insect monitoring" OR "smart trap"))	OR "pest monitoring" OR "pest detection" OR "pest identification" OR "pest classification" OR "insect monitoring" OR "smart trap")) AND TITLE-ABS-KEY(("integrated pest management" OR IPM OR "economic threshold" OR "decision support" OR forecasting OR "population prediction" OR "precision pest management" OR "biological control" OR "precision spraying"))	
IEEE Xplore	All Metadata: ("artificial intelligence" OR "machine learning" OR "deep learning" OR "computer vision" OR CNN OR YOLO OR "vision transformer" OR transformer OR "foundation model" OR "multimodal learning" OR "edge AI") AND All Metadata: ("insect pest" OR "crop pest" OR "agricultural pest" OR "pest monitoring" OR "pest detection" OR "pest identification" OR "pest classification" OR "insect monitoring" OR "smart trap")	All Metadata: ("artificial intelligence" OR "machine learning" OR "deep learning" OR "computer vision" OR CNN OR YOLO OR "vision transformer" OR transformer OR "foundation model" OR "multimodal learning" OR "edge AI") AND All Metadata: ("insect pest" OR "crop pest" OR "agricultural pest" OR "pest monitoring" OR "pest detection" OR "pest identification" OR "pest classification" OR "insect monitoring" OR "smart trap") AND All Metadata: ("integrated pest management" OR IPM OR "economic threshold" OR "decision support" OR forecasting OR "population prediction" OR "precision pest management" OR "biological control" OR "precision spraying")	Useful for embedded systems, edge AI, IoT, sensing, and computer-vision engineering literature.
ScienceDirect	("artificial intelligence" OR "machine learning" OR "deep learning" OR "computer vision" OR CNN OR YOLO OR "vision transformer" OR transformer OR "foundation model" OR "multimodal learning" OR "edge AI") AND ("insect pest" OR "crop pest" OR "agricultural pest" OR "pest monitoring" OR "pest detection" OR "pest identification" OR "pest classification" OR "insect monitoring" OR "smart trap")	("artificial intelligence" OR "machine learning" OR "deep learning" OR "computer vision" OR CNN OR YOLO OR "vision transformer" OR transformer OR "foundation model" OR "multimodal learning" OR "edge AI") AND ("insect pest" OR "crop pest" OR "agricultural pest" OR "pest monitoring" OR "pest detection" OR "pest identification" OR "pest classification" OR "insect monitoring" OR "smart trap") AND ("integrated pest management" OR IPM OR "economic threshold" OR "decision support" OR forecasting OR "population prediction" OR "precision pest management" OR "biological control" OR "precision spraying")	Useful for Computers and Electronics in Agriculture, Biosystems Engineering, Ecological Informatics, and related applied literature.
Supplementary methods	Backward and forward citation chaining from high-relevance reviews, benchmark datasets, and representative field deployments	Targeted searches for smart traps, ecological thresholds, forecasting, uncertainty, intervention, and outcome evidence	Document as supplementary discovery rather than merging with database hit counts.

Figure S1. Evidence-identification and transparency workflow

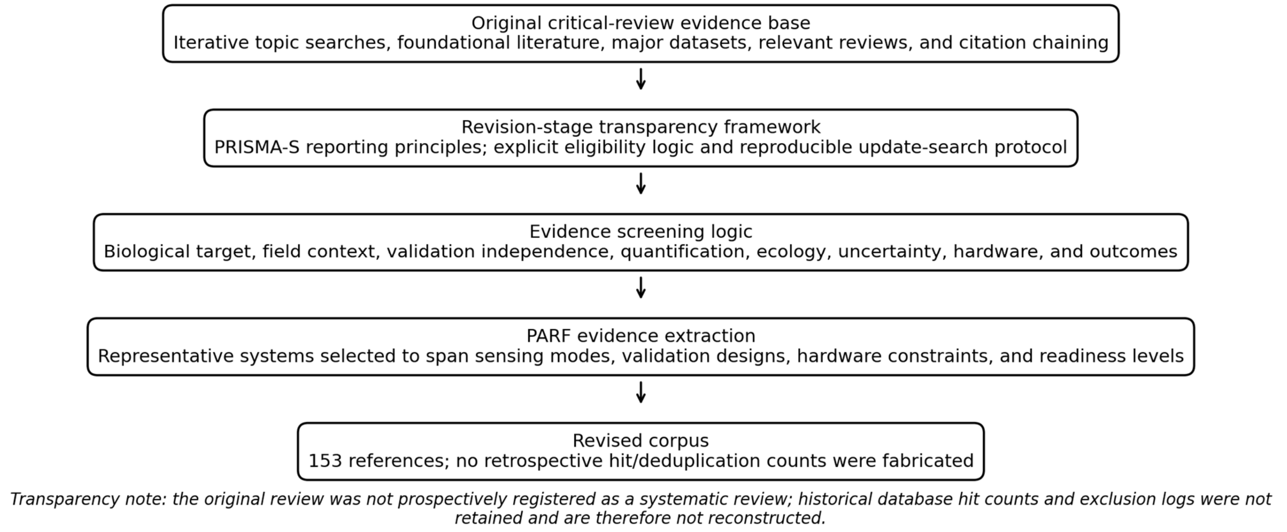

Figure S1. Workflow used to document evidence identification in the revised critical review. Because the original review was not prospectively registered as a systematic review, unverifiable retrospective screening counts are intentionally not supplied.

Table S2. Criterion-level source-audited evidence matrix for illustrative PARF assignments

Evidence-status codes: M = criterion explicitly established in the cited source; NR = the cited source explicitly does not report the item; NE = not established from the source-level evidence located/extracted in this review (NE does not imply that the original study necessarily lacked the evidence); N/A = criterion genuinely not applicable to the intended system function. An illustrative PARF level is assigned only when every required applicable criterion for that level is M. Source locators identify the section/table/figure used for the audit.

Study	Candidate function/level	Criterion	Status	Evidence extracted	Exact source locator	Final PARF
Wu et al. [22]	Recognition / PARF 1	Curated/controlled images and documented labels	M	75,222 images across 102 pest classes; bounding-box subset also released.	Dataset description/split; Table 1	1
Wu et al. [22]	Recognition / PARF 1	Internal validation design	M	45,095 train, 7,508 validation and 22,619 test images for classification; separate detection split.	Dataset split; Table 1	1
Wu et al. [22]	Field detection / PARF 2	Independent uncontrolled field acquisition/grouping	NE	Benchmark paper does not establish an operational field deployment separated by date/site/device/trap/region.	Benchmark dataset/evaluation sections	1

Study	Candidate function/level	Criterion	Status	Evidence extracted	Exact source locator	Final PARF
Sun et al. [27]	Recognition / PARF 1	Controlled acquisition	M	Six bark-beetle species were collected from pheromone traps; specimens were then randomly placed into the collection cup in the laboratory to simulate field traps.	In-trap image acquisition; Fig. 1; Table 1	1
Sun et al. [27]	Field detection / PARF 2	Uncontrolled field/trap acquisition plus independent grouping	NE	The audited acquisition was laboratory-simulated; an independent field acquisition date/site/device/trap grouping is not established by the cited evidence.	In-trap image acquisition	1
Sun et al. [27]	Supporting evidence	Embedded performance	M	Object-level AP 0.746; runtime 0.448 s on Jetson TX2 and 23.44 s on Raspberry Pi 3b.	Experiments and results	1
Geissmann et al. [96]	Automated monitoring / PARF 3	Repeated acquisition	M	Sticky Pi acquires sticky-card images every 20 min; 10 devices were deployed in two berry fields for 11 weeks.	Results - Sticky Pi device/platform; Results - Image processing; field deployment methods	3
Geissmann et al. [96]	Automated monitoring / PARF 3	Validated quantification and duplicate control	M	Cross-frame Siamese Insect Matcher tracks instances through image series, explicitly addressing repeated appearances of the same insect.	Fig. 2 and Image processing section	3
Geissmann et al. [96]	Automated monitoring / PARF 3	Operational duration / maintenance evidence	M	Approximately two weeks autonomy without solar; 11-week natural-condition deployment reported.	Results - Sticky Pi device and platform; field deployment methods	3
Diller et al. [100]	Field detection / PARF 2	Independent field evaluation	M	Field deployments in Greece, Austria, Italy, South Africa and Israel; >300 Israel/South Africa images not used in training were evaluated against entomologist inspection.	Methods - evaluation using field data; Results - Field validation; Table 1	2
Diller et al. [100]	Automated monitoring / PARF 3	Validated quantification / operation	M	Field-validation precision ranged from 86% to 97% for the three target fruit flies; continuous field operation ranged from 60 to 257 d; upload rates and absence of electronic malfunction were reported.	Results - McPhail e-trap function and attractiveness; Field validation; Fig. 4; Table 1	2
Diller et al. [100]	Automated monitoring / PARF 3	Temporal duplicate-control rule	NE	The source-level evidence audited here does not establish how the same trapped individual is prevented from being re-counted across repeated images.	Source-level audit of Methods/Results	2
Zhou et al. [104]	Field detection / PARF 2	Field device evaluation and quantitative comparison	M	Application tested on Samsung Galaxy S10e and LG Stylo 6; counting accuracy 78.1% for TSSM motiles and 75.3% for eggs; compared with manual counting.	Abstract; field-evaluation results	2
Zhou et al. [104]	Automated monitoring / PARF 3	Repeated acquisition and biologically justified monitoring window	NE	The audited evidence establishes on-demand field sampling/mapping but not a repeated automated monitoring window for the deployed application.	Abstract; field-evaluation results	2
Zhou et al. [104]	Automated monitoring / PARF 3	Uptime/maintenance	N/A	Continuous autonomous uptime is not the intended function of the manually initiated smartphone application.	Application design/Abstract	2
Zhou et al. [104]	Automated monitoring / PARF 3	Temporal duplicate control	N/A	The evaluated function is per-leaf on-demand counting rather than accumulation of repeated automated frames.	Application design/Abstract	2

Study	Candidate function/level	Criterion	Status	Evidence extracted	Exact source locator	Final PARF
Palma et al. [117]	Predictive intelligence / PARF 4	Biological context and sampling duration	M	Aphids in wheat, Coxilha, Rio Grande do Sul, Southern Brazil; weekly sampling from 2011 to 2019; 424 observations.	Section 2.5 (aphid case study)	4
Palma et al. [117]	Predictive intelligence / PARF 4	Prediction target and horizon	M	Outbreak defined using threshold x*=200 aphids across four traps; PBP predicts outbreak state at the subsequent monitoring sample. Weekly sampling makes the case-study horizon one monitoring interval (approximately one week).	Sections 2.1 and 2.5; Fig. 6	4
Palma et al. [117]	Predictive intelligence / PARF 4	Strict future-held-out evaluation	M	Initial 40%, 50%, 60%, 70% and 80% of the time series used for training and the later complement for testing.	Section 2.5; Table 2	4
Palma et al. [117]	Predictive intelligence / PARF 4	Relevant quantitative result	M	PBP test accuracy reported as 95% with the 80% chronological training split.	Table 2; Abstract	4
Palma et al. [117]	Predictive intelligence / PARF 4	Prospective intervention trial	N/A	A treatment trial is not a minimum criterion for PARF 4; intervention/outcome evidence belongs to higher functional levels.	PARF definition in present review	4
Bjerge et al. [110]	Automated monitoring / PARF 3	Repeated acquisition and monitoring duration	M	System tested during summer 2020 for 98 d and detected 2,994 insect tracks; summary data could be uploaded daily.	Abstract; Methods/Results	3
Bjerge et al. [110]	Automated monitoring / PARF 3	Validated quantification	M	Average precision 89% for eight species based on 504 manually verified tracks observed during 10 d.	Abstract; Results	3
Bjerge et al. [110]	Automated monitoring / PARF 3	Duplicate control	M	The ICT algorithm tracks individual insects and produces track-level identities/residence times rather than independent per-frame sums.	Methods; tracking workflow	3
Guo et al. [147]	Field detection / PARF 2	Uncontrolled field acquisition and quantitative validation	M	Yellow sticky traps and RGB cameras were installed in vegetable fields; automatic acquisition and YOLO-SIP counting were evaluated.	Abstract; experimental design/results	2
Guo et al. [147]	Field detection / PARF 2	Quantitative result	M	mAP 84.22%, aMAE 0.422 and aMSE 1.126.	Abstract; experimental results	2
Guo et al. [147]	Automated monitoring / PARF 3	Monitoring duration, duplicate control and uptime/maintenance	NE	These PARF 3 minimum items were not established with sufficient source-level evidence in the present audit and are not inferred as absent.	Source-level audit of cited article	2
Kapetas et al. [118]	Predictive intelligence / PARF 4	Biological context / sampling duration	M	Five sticky traps in cucumber greenhouse at UTH, Velestino, Greece; 220 images over 44 d (late October-early December 2023), near-daily on weekdays.	Section 2.1; Fig. 2	4
Kapetas et al. [118]	Predictive intelligence / PARF 4	Prediction target and horizon	M	Black-aphid population growth forecast over seven days using daily environmental variables and aphid counts.	Section 2.4	4
Kapetas et al. [118]	Predictive intelligence / PARF 4	Strict future-held-out evaluation	M	ARIMAX evaluation shown with sequential initial 80% training and final 20% testing.	Section 2.4; Fig. 6	4

Study	Candidate function/level	Criterion	Status	Evidence extracted	Exact source locator	Final PARF
Kapetas et al. [118]	Predictive intelligence / PARF 4	Model-specific quantitative result	M	ARIMAX MSE 75.61; the article reports this as an average deviation of approximately 8.61 insects/day.	Section 3 forecasting results; Fig. 6; Abstract	4
Kapetas et al. [118]	Predictive intelligence / PARF 4	Leave-one-out evaluation of ML models	NE	LOOCV does not by itself establish strict temporal future holdout; those ML results are not used as the basis for the PARF 4 assignment.	Section 2.4 / prediction-model evaluation	4
Kapetas et al. [118]	Predictive intelligence / PARF 4	Prospective intervention trial	N/A	A treatment trial is not a minimum criterion for PARF 4.	PARF definition in present review	4

Source-audit and PARF assignment note

For formal future applications, PARF assignments should be made by at least two independent evaluators using the same extraction fields and consensus resolution. This was a recommended implementation rule rather than a prospectively applied dual-review procedure for the original extraction in this critical review. For the present revision, the exemplar assignments were re-audited against explicit criterion-level source evidence and exact source locators, but no inter-rater reliability statistic is claimed. The assigned level is the highest functional claim for which all required applicable evidence is explicitly established; an NE item caps the level rather than being treated as satisfied or as proof of absence. N/A is used only when the criterion is genuinely outside the intended function.

Table S3. Descriptive bibliometric profile of the revised cited corpus

Bibliometric descriptors were extracted from the 153 references cited in the revised manuscript using publication year and journal/source title. The former title-keyword thematic classification was removed because composition of a selected citation list should not be interpreted as evidence of field-wide technology maturity. Table S3 therefore reports only descriptive publication-period counts and the most frequent journal titles in the cited corpus. The reference-level publication year, source and full citation used for these counts are supplied in machine-readable Supplementary Data S2.

Indicator class	Category	n	% of corpus	Interpretation
Publication period	<=2015	16	10.5%	Earlier/foundational references retained where conceptually necessary
Publication period	2016-2020	51	33.3%	Descriptive count within the selected cited corpus
Publication period	2021-2023	42	27.5%	Descriptive count within the selected cited corpus
Publication period	2024-2026	44	28.8%	Descriptive count within the selected cited corpus
Journal title	Computers and Electronics in Agriculture	23	15.0%	Most represented journal title in the selected cited corpus
Journal title	Sensors	8	5.2%	Second most represented journal title
Journal title	Agriculture	7	4.6%	Third most represented journal title
Journal title	Frontiers in Plant Science	6	3.9%	Fourth most represented journal title

Indicator class	Category	n	% of corpus	Interpretation
Journal title	Agronomy	5	3.3%	Fifth most represented journal title

Interpretation: These counts describe only the composition of the 153 references selected for this critical review. They are not estimates of field-wide prevalence, maturity or readiness and are not used to assign PARF levels. Readiness statements are based on the criterion-level study audit in Table S2 and the main-text PARF analysis.

Machine-Readable Supplementary Data: Supplementary Data S1 = PARF criterion-level evidence matrix (CSV); Supplementary Data S2 = reference-level publication-year/source data supporting the descriptive bibliometric profile (CSV).
